# Deep Convolutional Neural Networks for the Prediction of Molecular Properties: Challenges and Opportunities Connected to the Data

**DOI:** 10.1515/jib-2018-0065

**Published:** 2018-12-05

**Authors:** Niclas Ståhl, Göran Falkman, Alexander Karlsson, Gunnar Mathiason, Jonas Boström

**Affiliations:** School of Informatics, University of Skövde, Högskolevägen 28, SE 54145, Skövde, Sweden; School of Informatics, University of Skövde, Skövde, Sweden; Department of Medicinal Chemistry, CVMD iMED, AstraZeneca, Mölndal, Sweden

**Keywords:** Molecular property prediction, Deep learning, Unbalanced data, Side effects prediction

## Abstract

We present a flexible deep convolutional neural network method for the analysis of arbitrary sized graph structures representing molecules. This method, which makes use of the Lipinski RDKit module, an open-source cheminformatics software, enables the incorporation of any global molecular (such as molecular charge and molecular weight) and local (such as atom hybridization and bond orders) information. In this paper, we show that this method significantly outperforms another recently proposed method based on deep convolutional neural networks on several datasets that are studied. Several best practices for training deep convolutional neural networks on chemical datasets are also highlighted within the article, such as how to select the information to be included in the model, how to prevent overfitting and how unbalanced classes in the data can be handled.

## 1 Introduction

Discovering new chemical entities is a difficult and time-consuming endeavour [1], [2]. By using computers and algorithms the belief is to shorten the process from idea to launched drug. The field of cheminformatics is one area holding promise to enable faster and better decision-making, for example, by deriving new methods for automated predictions of molecular properties [3]. Many of the problems in cheminformatics are focused on how to predict properties of a molecule given the known values of these properties of several similar molecules. Due to the nature of these problems, cheminformatics is influenced by the development and trends in machine learning. One sub-field in machine learning that has expanded tremendously in the last years, mainly due to the success and advancement in several fields such as image recognition [4] and speech recognition [5], is *deep learning* [6]. Recently, deep learning has started to make its way into cheminformatics [7].

Several traditional machine learning algorithms have been applied for the prediction of molecular properties, such as *random forests* [8], *support vector machines* [9], *k-nearest-neighbours classification* [10] and *artificial neural networks* [3], [7], [11], [12], [13]. A major difficulty when applying machine learning algorithms on molecular data is that most machine learning algorithms require input of a fixed size, while molecules are of arbitrary size. To circumvent this problem, the most common approach is to pre-process the molecular data into chemical “fingerprints”; an abstract and fixed sized representations of structural features, enabling the application of selected machine learning algorithms [14]. This process is, for example used in Huuskonen et al. [15], Ma et al. [16], Ekins [17] and Mayr et al. [18]. However, once encoded as fingerprints, it is not trivial to trace back which part of the molecule gives rise to different effects, hampering interpretation.

In this paper we take a different approach and use deep convolutional neural networks (DCNN) on molecules represented as graphs as input. The same approach has previously been used by several other authors [19], [20], [21]. We show that the predictive power of such networks can be improved if more information about the atoms, bonds and molecules are added, for example chirality, bond type, the number of rotatable bonds and the mass of the molecule. While some, but not all, of the information used in this study has been used in previously presented papers concerning DCNN models, the reflection on how the selection of input information affects the result is negligible. To show that this cannot be overlooked we develop a flexible model where both global and local information easily can be incorporated or removed. Using this model, we explore how the predictive power varies when more information is gradually incorporated. It is worth to point out that this study does not aim to be an extensive search to find which information that contributes the most to the predictive power. Instead, we aim to show that different types of molecular information easily can be incorporated into a DCNN and that this can increase the predictive power of that particular network. Another issue that has not been given enough attention is the problem of unbalanced classes, a problem that often arises in chemical and medical datasets [22]. We show that by customising the training process of the presented DCNN to better handle unbalanced classes, the performance was significantly increased in one case.

There has not been any standardised way to measure the performance of predictive algorithms within cheminformatics against each other. However, most recently Wu et al. [21] made an effort and compiled several datasets that can be used as benchmarks for this purpose. The presented DCNN in this paper is evaluated on three different datasets, selected from this benchmark. A special focus is given to one of these datasets, in order to further understand the behaviour of DCNNs, namely the SIDER dataset, which originates from the SIDER database [23]. The reason that this dataset is selected for a closer study is that Wu et al. [21] acknowledge SIDER to be a dataset where it is difficult to achieve a high predictive accuracy. Surprisingly, while being one of the best performing methods on all other datasets in Wu et al. [21], their presented DCNN performed very poorly on the SIDER dataset and was outperformed by traditional methods such as *random forest* and *logistic regression*. However, both of these methods are less flexible and requires the use of chemical fingerprints. Therefore, in order to be able to improve further DCNN methods, on the SIDER dataset and datasets with similar properties, we argue that there is a great need to study and find the reasons for why the DCNN method performed so poorly in this case. While Wu et al. [21] argued that the reason is that the dataset consists of biological molecules, we believe that this only partly explains the low average area under the receiver operating characteristic curves AUC-ROC value [24]. Instead we show that a part of the poor performance of the DCNN is caused by the imbalance in the data for some of the side effects. By further investigating the reasons behind the bad performance of DCNNs on the SIDER dataset, we hope to show best practices for how deep learning methods can be improved in the future.

## 2 Deep Learning in Cheminformatics

While neural networks have been used in cheminformatics for several decades, deep learning has just recently made its way into this field [7]. Several groups have applied deep neural networks on molecular fingerprints for the prediction of many different properties, including toxicity [18] and solubility [15]. One of the most famous applications of neural networks in cheminformatics is the work of Dahl et al. [25], which won the Merck Molecular Activity Challenge, a competition where researchers were invited to predict how small molecules acted on 15 different target molecules. However, deep feed forward neural networks like these are limited to input of fixed size. Since molecules can have an arbitrary size, some feature extraction must be conducted to reduce the molecule to a fixed set of values before the deep neural networks can be applied. This is most commonly achieved by the creation of a fixed sized vector of descriptors for each molecule [13], leading to information loss. Several authors overcome this flaw by either using a recurrent neural network (RNN) or a DCNN. Lusci et al. [26] do for example use a RNN for the prediction of aqueous solubility. In their work, Lusci et al. [26] first convert the molecular structure into directed acyclic graphs (DAGs). Each of these DAGs are traversed by a RNN, giving a vector representation for each DAG. These vectors are then summarized into a vector representing molecular properties, from which a prediction of the solubility can be done. Xu et al. [27] used the same method to predict if a molecule would cause liver injury or not.

Another approach that has been used by other authors is to use a DCNN for the prediction of molecular properties. These authors assume that low level features in the molecule will emerge due to local interactions between neighbouring atoms in the same way as low level image features, such as edges, emerges due to interactions between neighbouring pixels in an image [4]. Wallach et al. [28], for example, apply a three dimensional convolutional neural network on the spatial structure of molecules. Duvenaud et al. [19] Kearnes et al. [20] and Wu et al. [21] use another approach and represent molecules as graphs where nodes correspond to atoms and the edges to bonds. Several types of convolutional neural networks, which use different type of molecular information and with different purposes, are then applied to these graphs representing molecules. One example of this is Duvenaud et al. [19], which use a DCNN that uses atom properties (such as the hybridization type of the atom and if the atom is in a ring or not) and bond properties (such as the type of the bond) to automatically generate fingerprints. Kearnes et al. [20] use a different type of DCNN, which consists of so called “weave modules”, where information is transferred between different atoms. Wu et al. [21] present several chemical datasets for benchmarking of machine learning algorithms. The performance of several algorithms, among them a DCNN for graphs, is evaluated on each dataset. However, even though these authors do not use the same atom and bond information in their networks, none of them reflects on how this affects the result. This is important since different molecular properties may emerge from completely different molecular attributes. Thus the information included in a model may be crucial for its success. Deep learning has been used for more tasks in cheminformatics than just property prediction. Segler et al. [29] do, for example, use RNNs as generative models to generate new SMILES strings, a way to store molecules in form of a line notation [30], (in the same way as Graves [31] used recurrent neural networks to generate text). These generated SMILES strings were then used to find novel drug candidates.

In the next section, our proposed network architecture is presented. It is similar to the architectures presented by Duvenaud et al. [19] and Wu et al. [21]. In addition, our architecture incorporates several other advances within deep learning, such as residual learning [32] and dropout layers [33].

## 3 Proposed Model

**Figure 1:**
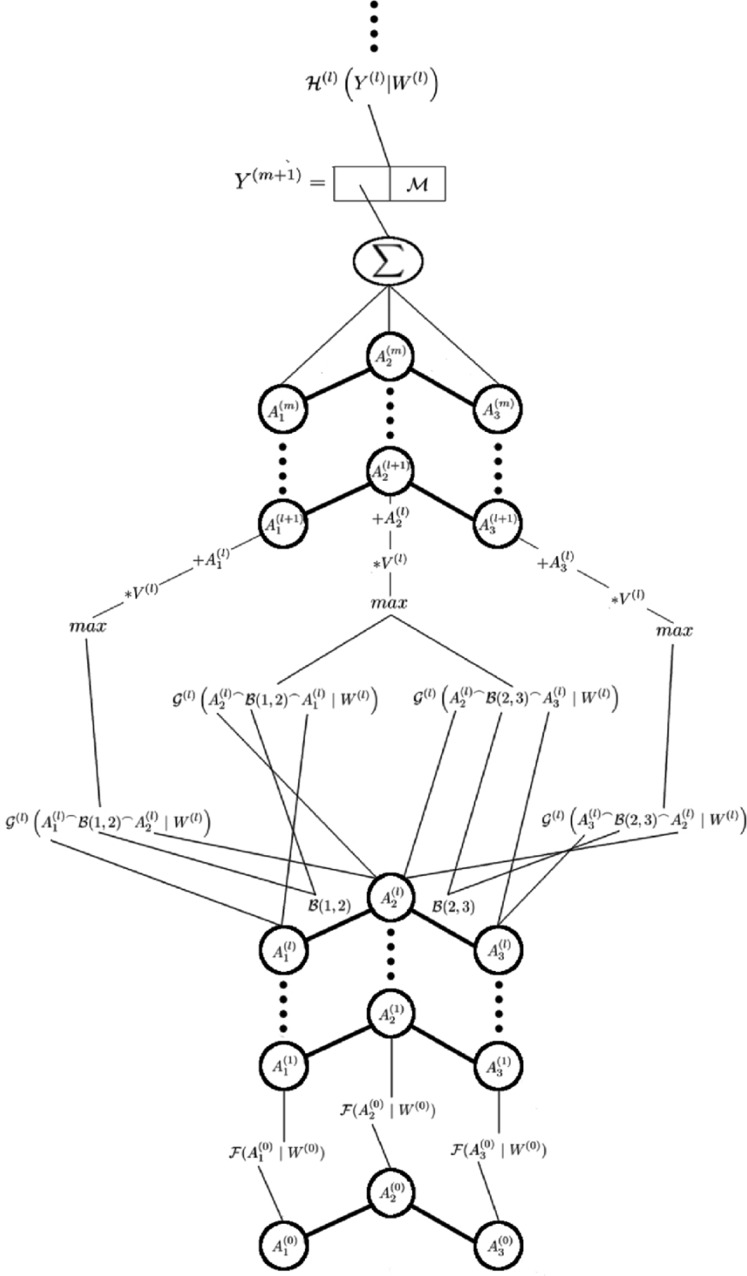
A graphical representation of the proposed model. This figure shows an example where the model is applied to a molecule consisting of three atoms. The equations governing the inner workings of this model are described in equations (1) to (5).

The model architecture, shown in Figure 1, is a slightly modified version of the DCNN presented by Duvenaud et al. [19] and Wu et al. [21]. This model is also inspired by the works of He et al. [32] and uses residual learning, something Duvenaud et al. [19] and Wu et al. [21] did not. The first step in our model is to calculate the initial hidden representation of each atom. Let $A_{i}^{(0)}$ be a vector consisting of all information that is extracted from atom *i*. Here the superscript represents the layer number. The contents of this vector vary between the different experiments, described in Section 4.2.

In the first and most simplistic experiment, $A_{i}^{(0)}$ corresponds to an “one hot” encoded vector, that is a vector where each elemental type corresponds to a given position in the vector. The value at the position of the elemental type of atom *i* is 1 and and the value of all other positions are 0. In later experiments, $A_{i}^{(0)}$ the “one hot” encoded vector is appended with real values, representing attributes such as the number of other atoms atom *i* binds to.

Let $A_{i}^{(1)}$ be the vector representing the first hidden state of atom *i*, given by

(1) $$\begin{aligned}A_{i}^{(1)}&=\mathcal{F}\left(A_{i}^{(0)}\ ;\ W^{(0)}\right),\end{aligned}$$

where *ℱ* is an arbitrary activation function and *W*^(0)^ is its parameters. Due to the conventions of the field, *ℱ* will be defined as the leaky ReLU function [34] in the rest of this paper due to current conventions. The next operation in the model is the convolutional steps. In these steps, information is transmitted between atoms and their neighbours. The steps can be expressed as

(2) $$C_{i,j}^{(l)}=\mathcal{G}^{(l)}\left(A_{i}^{(l)\frown}\mathcal{B}(i,j)^{\frown}A_{j}^{(l)}\ ;\ W^{(l)}\right),$$

(3) $$A_{i}^{(l+1)}=A_{i}^{(l)}+V^{(l)}\left[\max\limits_{j\in neighbourhood(i)}C_{i,j,1}^{(l)},\dots,\max\limits_{j\in neighbourhood(i)}C_{i,j,k}^{(l)}\right].$$

Equation (2) calculates how atom *i* is affected by having atom *j* in its neighbourhood. The function *ℬ* in equation (2) extracts information about the interactions between atom *i* and *j*, for example if there is a single, double, or triple bond between them. The ^⌢^ operator represents concatenation of two vectors. $\mathcal{G}^{(l)}$ is an arbitrary activation function, witch is parametrised by *W*^(l)^, and as with the function *ℱ* it will be defined as the leaky ReLU function in the rest of this paper. The hidden representation in each atom is then updated as described by equation (3). Here the *max* function is applied element-wise to every element in the vectors and the results are then multiplied with the square matrix *V*^(l)^, which is one of the parameters that the model later on will learn. Following the method used by He et al. [32], the result of the matrix multiplication is then added to the previous hidden representation of atom *i*. It has been shown that DCNNs having such short-cut connections, a connection where the signal is passed forward without any modification, are more stable than DCNNs not having such connections [32].

After *m* convolutional and max-pooling steps a global representation for the molecule is calculated. This is done by summing up the representation of all atoms and also adding global molecular properties. This can be expressed as

(4) $$\begin{aligned}Y^{(m+1)}=\sum_{i}\left(A_{i}^{(m)}\right)^{\frown}\mathcal{M},\end{aligned}$$

where *ℳ* is a vector of the selected molecular properties. Which molecular properties that are selected is described in Section 4.2. The use of the summation is arbitrary and may be replaced by any function that results in a fixed sized vector. However, this would most likely affect the result of the model. This gives the first hidden representation for the molecule. Standard hidden layers, which are defined as

(5) $$\begin{aligned}Y^{(l+1)}=\mathcal{H}^{(l)}\left(Y^{(l)};W^{(l)}\right)\end{aligned}$$

are then applied to this representation, which finally gives the predicted molecular properties. The *ℋ* in equation (5) represents an arbitrary activation function. In this paper the leaky ReLU function will be used for $\mathcal{H}^{(l)}$ except in the final layer where the sigmoid function will be used instead. The sigmoid function is selected here since we want the output of our model to be in the range of 0–1.

In one of the experiments, conducted in this paper, the problem of class imbalance is addressed. This is done by customizing the loss function of the network. Instead of using the standard binary cross entropy error (described in equation (6)), which is a common practice, a weighted version is used. The weighted version, as described in equation (7), takes the distribution of each class into account and thus rare instances will impact the loss much more. The standard cross entropy is defined as:

(6) $$\sum_{i=0}^{n}\sum_{j=0}^{c}-y_{i,j}log(\hat{y}_{i,j})-(1-y_{i,j})log(1-\hat{y}_{i,j}),$$

while the weighted version is defined as;

(7) $$\sum_{i=0}^{n}\sum_{j=0}^{c}-\left(\sum_{i=0}^{n}\frac{1-y_{i,j}}{n}\right)y_{i,j}log(\hat{y}_{i,j})-\left(\sum_{i=0}^{n}\frac{y_{i,j}}{n}\right)(1-y_{i,j})log(1-\hat{y}_{i,j}).$$

In equations (6) and (7), *n* is the number of samples in the dataset, *c* is the number of binary classes to predict, *y_i,j_* is the true class *j* of sample *i* and $\hat{y}_{i,j}$ is the predicted probability that class *j* is equal to 1 for sample *i*. The definition of equation (7) is selected in such a way that rare samples, that are miss classified, contribute much more to the total loss than those that are common.

## 4 Experiments

In this section, the experiments conducted using the model from the previous section, are described. To demonstrate the improvements that can be achieved by adding more information to the model, we conduct several experiments, each with gradually increasing level of provided molecular information. In the final experiment, a custom version of the training method is used to increase the predictive power for unbalanced classes.

### 4.1 Data

An investigation of the model, presented in the previous section, is conducted on three different datasets: SIDER, TOX21 and ClinTox. The molecules are stored as SMILES strings [30] and are read into the program and converted into a graph structure using the cheminformatics open source software RDKit [35] and the Lipinski module. RDKit is also used to extract information about the atoms, bonds and molecules, such as chirality and molecular weight. To be able to do a comparison between the achieved results presented in this paper and in the benchmark presented by Wu et al. [21], the dataset is split into training, validation, and test set depending on index, that is the order the samples appear in the dataset. As in the benchmark, 80% of the molecules are used to train the model, 10% as a validation set to find the best configuration, and the final 10% are used for testing.

#### 4.1.1 SIDER

The SIDER1https://github.com/deepchem/deepchem/blob/master/examples/sider dataset contains information on molecules, marketed as medicines, and their recorded side effects. This data originates from the SIDER database [23] and do originally consist of 1430 molecules and 5868 different types of side effects. However, in the dataset presented by Wu et al. [21], similar side effects are grouped together, leaving the dataset with 28 groups of side effects. Some of these side effects are very rare, and the most uncommon only occur in around 1.5% of all samples. Other side effects are common and several of them occur in more than 90% of the samples. Thus, many of the classes in the dataset are very unbalanced.

#### 4.1.2 TOX21

The TOX21, dataset2https://github.com/deepchem/deepchem/blob/master/datasets/ was collected in the “Toxicology in the 21^st^ Century” initiative which aimed to create a public dataset for the toxicity of compounds [36]. There are 8014 compounds in this dataset and beside information of the structure of the molecules it contains qualitative toxicity measurements on 12 different targets.

#### 4.1.3 ClinTOX

ClinTox3https://github.com/deepchem/deepchem/blob/master/examples/clintox/datasets/ is a dataset that was introduced by Wu et al. [21], in effort to gather a benchmark to measure the performance of machine learning algorithms within chemistry. ClinTOX contains both drugs that were approved by the US food and drug administration (FDA) and those that failed their clinical trials for toxicity reasons. There are a total number of 1491 drug compounds in the dataset and the toxicity (binary) and FDA approval status is recorded for each compound.

### 4.2 Experiments

In all experiments, the performance of the presented model is compared to the benchmark presented by [21]. In these experiments, it is investigated how the performance of the model is affected when additional information, about the molecule and its atoms, is added to the model. To this end, we conduct five different experiments, each using more information about the molecules than the previous experiment. In the most simplistic experiment only the elemental type of the atom is used. In the latter experiments, more information is used and information concerning the bond and the full molecular structure is also incorporated into the model. In the final experiment, the training method is customised to further handle the unbalanced classes in the SIDER dataset. The details of how this is done is described in the next Section. Five different experimental set-ups are used to show how the performance of the model is affected when more information is added. One additional set-up is used to show how the problem of unbalanced classes can be handled. The six set-ups used, are the following:

1. The elemental type of each atom.
2. The elemental type of each atom and its hybridization type.
3. The elemental type of each atom, its hybridization type and the type of each bond.
4. Information concerning the atom, including elemental and hybridization type, chirality, the number of hydrogen atoms the atom binds to, if the atom is in a ring and if that ring is an aromatic ring. Information about the type of bond will also be used.
5. The same information as used in set-up 4, adding information concerning the complete molecule. Information such as weight, charge and the number of rotatable bonds in the molecule.
6. The same information as used in set-up 5, but to increase the performance for skewed classes, the customised loss function described in equation (7) is used.

### 4.3 Implementation

The model described earlier in Section 3 is implemented in Theano [37]. To perform a good comparison between our model and the model presented in Wu et al. [21], we choose to use an as similar network architecture as possible. Therefore we choose to have two convolutional layers, each with 64 neurons. These layers are then followed by a single fully connected layer, with 128 neurons. Between each layer in our model we use a 10% dropout rate, a method described by Srivastava et al. [33], to reduce the overfitting of the model. The models are trained by minimizing the cross entropy error, described in equation (6), between the predicted values and the real measured values for all molecules in the dataset. This is achieved by optimizing the values of all free parameters (*W*^(l)^ and *V*^(l)^) in the model using the ADAM optimization algorithm [38].

For each experimental set-up, ten networks with different initial parameters are trained. Each network is trained for 200 epochs using a batch size of 20 examples. The networks are evaluated after each epoch and the configurations that achieves the best results on the validation data are later used to evaluate the performance on the test data. The performance of the networks is evaluated in the same way as in Wu et al. [21] by calculating the AUC-ROC. To do a comparison, the final performance measure of each experiment is calculated as the mean value of the AUC-ROC for the different predicted variables, averaged over the ten different networks.

### 4.4 Results

**Figure 2:**
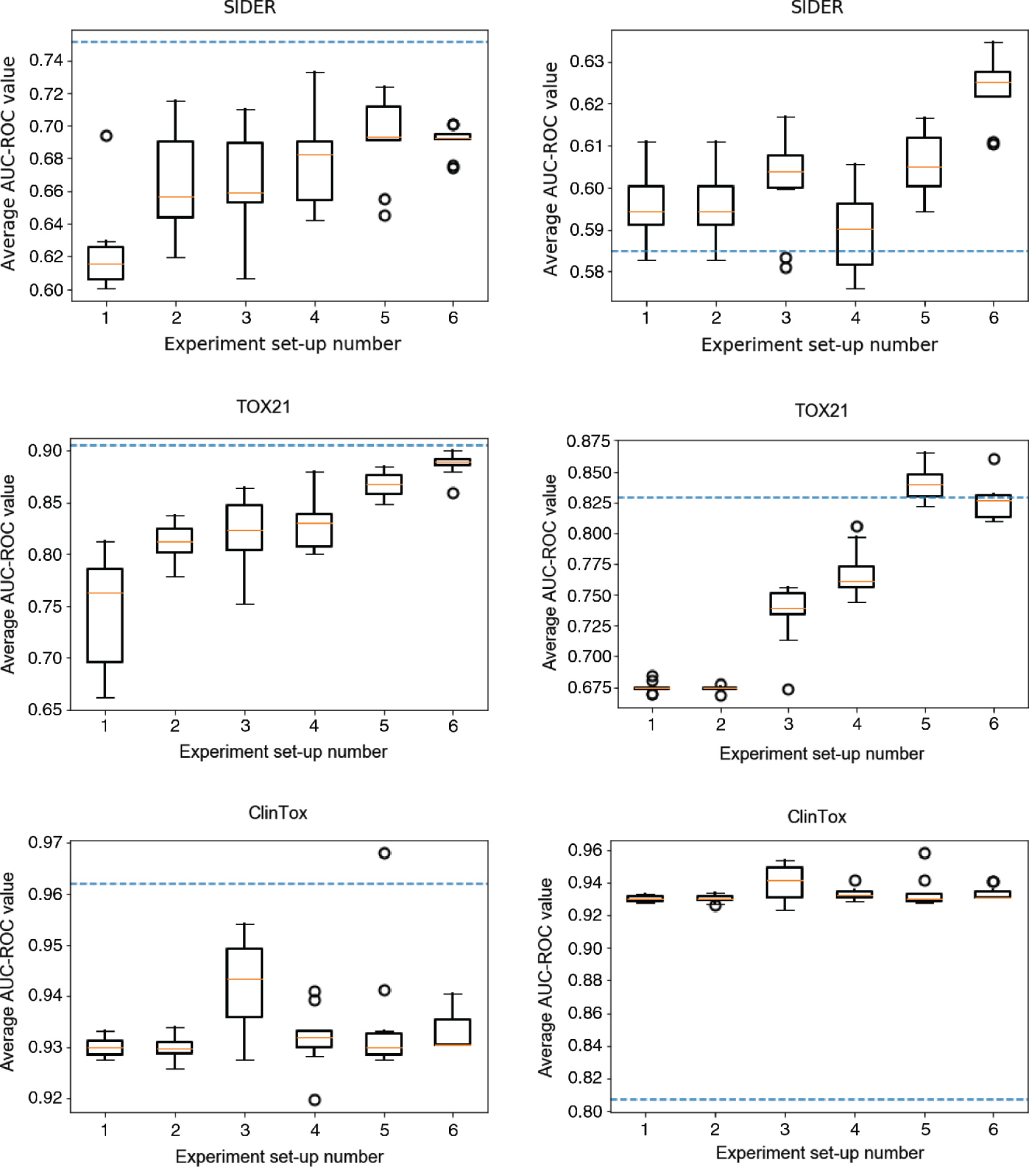
The distribution of the achieved AUC-ROC values averaged over all target variables for each experimental set-up. The blue dashed line represents the result achieved by the GCNN presented by Wu et al. [21]. The left plot is the achieved results on the training data and the right plot is the achieved results on the test data.

**Figure 3:**
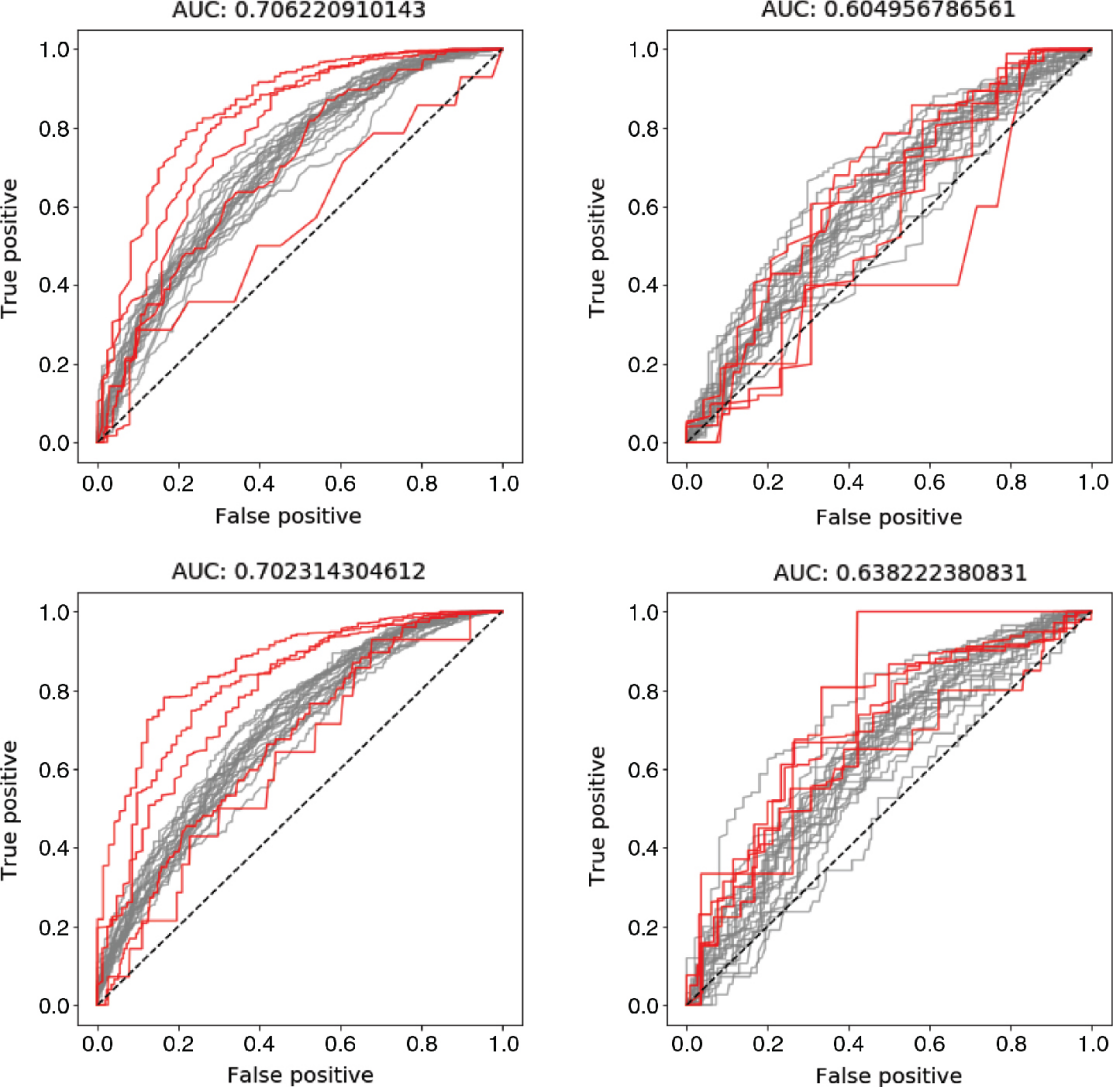
The ROC curves for each target variable from a model trained using the information described in experiment set-up 5 and set-up 6 for the SIDER dataset. The ROC curves for the five most unbalanced classes are highlighted in red, in order to show how the performance is affected by the weighting of the loss function. The top row shows results achieved using experiment set-up 5 and the bottom row results from experimental set-up 6. The left column shows the ROC curves for the training data and the right column for the test data. Note that the results for the test data are almost similar for the two set-ups while the performance for the test data increased in set-up 6. This is due to a better separation of the unbalanced classes, something that otherwise will bring down the mean AUC-ROC value.

In this section the results from the presented models and the described experiments are compared with the results from the Graph Convolution Network presented by Wu et al. [21]. The results of this comparison are shown in Table 1– Table 3. In these tables it is shown that the presented model outperformed the Graph Convolution Network presented by Wu et al. [21] when the model had access to the most available information. A graphical representation of these results are shown in Figure 2. The differences to the result achieved by Wu et al. [21] are also shown in these graphs. For two of the datasets, SIDER and TOX21, it was a clear difference in performance between the experiments when only atomic levelled features were used compared to when global features were added. In the case with the third dataset, the model achieved a high AUC-ROC value already in the first experiment, and hence the possibility to improve this result was very limited. The balancing of the classes had a great impact on the obtained results when the SIDER dataset was studied. In this case, the most significant improvement of the model was when the unbalance among the classes was handled by a weighted loss function. Thus, a large improvement could be achieved by handling the problem of unbalanced classes, resulting in some rare samples being correctly classified. This is shown in Figure 3, where typical views of how the individual ROC curve may look for each target variable for the training and test data in experiment set-up 5 and 6. In this Figure, it can be seen that some of the target variables, that are unbalanced, contribute very negatively to the mean by having low AUC-ROC values in experiment set-up 5. However, when the unbalance is handled this is no longer the case.

Using a *t*-test it was shown that the presented model, using the experimental set-up 5 with the most information available, preformed significantly better than the model presented by Wu et al. [21] on two of the studied datasets. The *p*-value yielded by these two *t*-tests were less than 0.0001. For the third dataset the model still performed better, but the difference in performance was not significant.

## 5 Discussion

The results of the model presented in Section 3 were significantly better than the results previously obtained on the same dataset with the use of a DCNN [21] in two out of three cases. However, in the experiments with the SIDER dataset there are still other models, such as random forest, achieving even better results than our presented model. In this case, it is most likely that the result could be further improved by finding a better network architecture for the presented DCNN model, something that Wu et al. [21] also point out. There is also room for improvement given how evaluation of the models is conducted in relation to how the models are trained. The objective of the evaluation metrics are for example not the same as the objective against which the training procedure optimizes the models.

The information about the molecules that was used in the conducted experiments is just a small subset of all information that can be extracted from each molecule, bond and atom. There are also a large number of network architectures that were not tested in this study. Therefore there should be an opportunity to achieve a better result by adding even more molecular information to the model or to finding a better network architecture. The optimal network architecture would also most likely differ between the experiments that are conducted. The more information that is used, the larger network is needed to make use of all information.

Any deep neural network would theoretically perform as good as or better than before when more information is added to the network. The reason for this is that the network can always learn to ignore the additional information. This argumentation follows the same chain of reasoning as He et al. [32] used when arguing for why adding more layers to a deep neural network should theoretically always improve the performance of the network. However, while it is possible to argue that adding more information to the model always improves the predictive power, this is not always true for practical experiments. If too much irrelevant information is added to the network there is always a risk that the relevant information will “drown” in the heap of useless information and thus it would be difficult to correctly train the network. Another problem that may occur when too much information is added to the model is that the dimensionality of the input data increases, which in its turn increases the risk of overfitting. Experiment set-up 4 for the SIDER dataset is a good example of this. When more information is added, in comparison to previous experiments, the AUC-ROC increases for the training data while the AUC-ROC decreases for the test data, as shown in Figure 2.

Some of the information that is added to the model is redundant and it should be possible for the network to calculate it from the rest of the information. An example of this is the molecular weight, which could be calculated knowing the elemental type of each atom. However, adding the information facilitates the networks ability to learn other useful features. Having a flexible model where both local and global information, with a minimal effort, can either be added or removed from the model lets domain experts customize models depending on problem.

Besides that the presented model performed better than the model by Wu et al. [21] it also suffers less from overfitting. This is due to the introduction of dropout and residual learning to the model. These methods makes the training and testing stable and should thus be considered when developing new models.

By customizing the loss function that is optimized during training of the model, we show that the performance of the presented model was greatly improved for the SIDER dataset. However, when introducing the weighting among the different classes, we assume that the dataset captures the class distribution among new samples, an assumption that may not always be true. Thus the improvements shown in this paper can not be generalized to new datasets, where the distribution between the training and test set differs. This can be seen in Figure 3 where the curves with the lowest AUC-value are those representing the prediction of variables in the very unbalanced classes. The reason that made us believe that the classes in the dataset are unbalanced was that Wu et al. [21] achieved a better result with the random forest algorithm, compared to both their and our DCNN method. We hypothesised that this is a sign that the dataset contains unbalanced classes, since the random forest algorithm is much more robust to this [39].

The problem with unbalanced classes is often present in chemical and medical datasets [40]. However, how the model is affected by this is not always considered. The difference in how this is handled in the model is highly dependent on the objective and the metric used as optimization objective during training. Some metrics, such as the percentage of correctly classified samples and the cross-entropy, only measure the classification accuracy of the model. Thus, there are no great loss in classifying rare samples wrong. However, other metrics such as the AUC-ROC and the *F*-score measures relative scores per class. Therefore, if any of these metrics are used, it is very important to classify rare instances correctly, since these greatly affects the metric. The real problematic case is when different metrics are used for training and evaluating a model, for example when minimizing the cross entropy error during training and then using AUC-ROC as the evaluation metric. Therefore, it is of great importance to consider not only the selection of models, but also the selection of metrics. To avoid this problem we suggest that the same objective metric should be used when both training and evaluating the model. Besides this, it is also important to reflect on how the results are affected by the selection of metrics.

## 6 Conclusion

We present a deep convolutional neural network model that performs significantly better than the DCNN model presented in [21] on two of the three studied datasets, SIDER and Clintox. Our model, which uses the open-source cheminformatics tool-kit RDkit, is flexible and makes it easy to add further molecular properties to the model. It is possible to add or remove any information about atom, bond or molecule from the molecule. Using this model, we show that adding more information is mostly beneficial. However, we show and discuss that there is no guarantee that a model will perform better when more information is added. Therefore, more work is needed to find which information that should be incorporated into the model and how to select the network architecture to achieve the optimal result. The optimal information used in the model is most likely very dependent on the dataset and problem. Thus it is of great utility to have a model where it is easy to include all sorts of information regarding molecules relevant to the given problem. As expected, the best performance of the model was obtained when the most molecular information was incorporated into the model. More surprising is that the performance of the model was almost the same when only the elemental types of the atoms were used.

In the paper, we point out some weaknesses of current deep learning models used in cheminformatics, including the presented model. These issues need to be addressed to further advance the use of deep learning within the field of cheminformatics. By selecting datasets from the benchmarks proposed by [21], this work is also a step in the right direction to form a standardized way to evaluate machine learning algorithms in the field of cheminformatics.

This paper highlights several best practices of how to design DCNNs to increase the performance on a given dataset. This includes preventing overfitting by adding dropout and residual learning to the network. It is also shown that selecting more molecular information increases the performance. For unbalanced datasets, such as SIDER, a large improvement in performance also came from handling the unbalance in some of the classes.

## Appendix

In this section the mean AUC-ROC values, achieved by the presented model in all described experimental set-ups and for all datasets, are presented. These results are also compared with the results in Wu et al. [21] which are shown in the last row of each Table.

**Table 1:** Results for the SIDER dataset.

SIDER	Training	Validation	Testing
Experiment set-up 1	0.622	0.554	0.595
Experiment set-up 2	0.663	0.558	0.595
Experiment set-up 3	0.666	0.560	0.602
Experiment set-up 4	0.681	0.586	0.589
Experiment set-up 5	0.693	0.589	0.605
Experiment set-up 6	0.691	0.594	0.623
Graph convolution [21]	0.751	0.613	0.585

**Table 2:** Results for the TOX21 dataset.

TOX21	Training	Validation	Testing
Experimental set-up 1	0.745	0.696	0.675
Experimental set-up 2	0.812	0.772	0.674
Experimental set-up 3	0.82	0.801	0.735
Experimental set-up 4	0.829	0.795	0.768
Experimental set-up 5	0.867	0.847	0.839
Experimental set-up 6	0.887	0.861	0.825
Graph convolution [21]	0.905	0.825	0.829

**Table 3:** Result for the ClinTox dataset.

ClinTox	Training	Validation	Testing
Experimental set-up 1	0.932	0.93	0.921
Experimental set-up 2	0.931	0.929	0.927
Experimental set-up 3	0.942	0.942	0.94
Experimental set-up 4	0.932	0.932	0.933
Experimental set-up 5	0.935	0.928	0.934
Experimental set-up 6	0.933	0.929	0.931
Graph convolution [21]	0.962	0.920	0.807
